# The Effect of Different Types of Laryngeal Mask Airways on Sound Quality: A Prospective Randomized Study

**DOI:** 10.7759/cureus.19056

**Published:** 2021-10-26

**Authors:** Musa Zengin, Arzu Akdaglı Ekici, Guvenc Dogan, Dogan Atan, Ali Alagoz

**Affiliations:** 1 Anesthesiology and Reanimation, University of Health Sciences, Ankara Atatürk Chest Diseases and Thoracic Surgery Training and Research Hospital, Ankara, TUR; 2 Anesthesiology and Reanimation, Hitit University, Erol Olcok Training and Research Hospital, Corum, TUR; 3 Otorhinolaryngology, Lokman Hekim University Faculty of Medicine, Ankara, TUR

**Keywords:** voice performance, shimmer analysis, laryngeal mask airways, i-gel, general anesthesia

## Abstract

Background

Although the deterioration in sound quality is not as much as endotracheal intubation, it can also be seen after the use of laryngeal mask airway (LMA). The aim of this study is to investigate the effects of different LMA types on voice performance.

Methods

This study included 88 patients aged 18-80 years, whose surgical procedure was not planned to take longer than 120 minutes. In the acoustic voice analysis, F0, jitter%, and shimmer% were examined. In addition, the Voice Handicap Index (VHI)-30 questionnaire has completed an evaluation of voice quality. The patients were randomly divided into two groups (I-gel LMA [n=44]; Classic LMA [n=44]) according to the closed envelope method.

Results

A total of 88 patients were included in the study. Demographic data of the patients were statistically similar between the groups (p > 0.05). The changes in the preoperative and postoperative F0, jitter, and VHI-30 values in the I-gel group were statistically significant (p: 0.002, p: 0.001, p < 0.001). Shimmer values were not significantly different (p: 0.762). In the classical LMA group, preoperative and postoperative F0, jitter, shimmer, and VHI-30 values were statistically significantly different (p: 0.001, p: 0.012, p: 0.036, p < 0.001).

Conclusion

I-gel LMA and classic LMA negatively affect voice performance in the preoperative and postoperative periods. This situation was also observed in the VHI-30 index test. However, when this situation was evaluated in terms of shimmer analysis, the decrease in voice quality in the early postoperative period was more limited in the I-gel group.

## Introduction

The classic laryngeal mask airway (LMA) started a new era in the practice of airway management and is now routinely utilized in clinical anesthesia [1]. Nevertheless, there are limitations associated with the classic LMA, such as controlled ventilation being relatively contraindicated and its unsuitability for patients at risk of aspiration [1,2]. To solve these problems, second-generation supraglottic airway devices (SADs) were designed. Newer SADs have additional safety features developed for the esophageal and pharyngeal seals. Aspiration and ventilation of the stomach can be achieved with a channel that enables the passage of the nasogastric tube. In this way, gastric entry is provided and the risk of aspiration is minimized [1].

With the development of new types of LMAs, SADs such as ProSeal LMA (PLMA) have become more preferred by anesthetists instead of first-generation SADs. This may be related to better airway sealing pressures in newer generation SADs and allowing the placement of a drainage tube to ventilate the stomach [3]. However, the newer SAD, I-gel airway, may provide more advantages in terms of safety and comfort by providing adequate airway sealing without the need for an inflatable cuff [4,5].

Laryngoscopy and endotracheal intubation (ETI) develop a sympathetic reflex response based on the mechanical stimulation of the larynx and trachea [6]. It is also known that ETI produces more postoperative pharyngolaryngeal morbidity, including voice change, compared to laryngeal masks [3,7]. Studies comparing the incidence of postoperative sore throat and hoarseness with the ETI and LMA have shown that these side effects are significantly higher in patients with an ETI preferred for general anesthesia than in those using LMA [8-10]. Therefore, LMA is recommended as an important alternative to endotracheal intubation, especially for professional voice users, unless contraindicated [3,11].

Although there are many studies regarding the effect of ETI and LMA on voice quality, it is not valid for different LMA types [3,7]. We hypothesized that I-gel LMA may cause more limited hoarseness and impairment of vocal functions than classical LMAs. Our aim in this study is to investigate the effects of different LMA types on voice performance.

## Materials and methods

This study was conducted between March 2019 and July 2019 at a tertiary education and research hospital. This study was approved by Hitit University Ethics Committee, and informed consent was obtained from the patients (Date: 01.03.2019, ID: 2019-93). This study included 88 patients aged 18-80 years, conforming to the ASA I-II class according to the classification of the American Society of Anesthesiologists (ASA), who was scheduled to undergo surgery that will not last more than 120 minutes with the patients in the supine position, except an airway or laparoscopic surgery. Nasopharynx and larynx examinations of the patients were performed before and after surgery by an otolaryngologist.

Patients with lung disease, obese (BMI > 35 kg/m2), pregnant women, those with a history of gastroesophageal reflux and suspected difficult airway findings (mouth opening < 2.5 cm, Mallampati score > 2, sternomental distance < 12.5 cm, thyromental distance < 6 cm) and neck circumference > 40 cm) were excluded from the study. In addition, patients with a high risk of aspiration pneumonia, using inhaled steroids, airway obstruction due to upper respiratory tract pathology, and patients with active infection were not included in the study. The patients were randomly divided into two groups (I-gel LMA [n = 44]; Classic LMA [n = 44]) according to the closed envelope method. During the operation, premedication was not performed in the patients according to the general anesthesia protocol. Patients were monitored in the operating room according to the standard ASA guidelines. Anesthesia was induced with propofol 3 mg.kg-1 and fentanyl 2 mcg.kg-1. Patients were ventilated with 100% O2 for three minutes at a flow rate of 6 lt.min-1, and appropriate LMA, that conforms to the patient’s age and body weight, placed by an expert anesthesiologist in a single attempt. The visualization of the capnography wave and bilateral chest expansion during manual ventilation was considered as the indicators of effective ventilation. Anesthesia was maintained with 1% to 2% sevoflurane in 50%/50% O2/air mixture and remifentanil infusion by controlled mechanical ventilation to achieve a tidal volume of 6-8 mL.kg-1 and respiratory rate of 12 breaths per minute in all patients. The LMA was removed after spontaneous breathing returned in all the groups.

Acoustic voice analysis was performed on the patients preoperatively and in the first 48 hours postoperatively. In the acoustic voice analysis, F0, jitter%, and shimmer% were examined. In addition, the Voice Handicap Index (VHI)-30 questionnaire was completed under the supervision of a physician for the subjective evaluation of voice quality. The effect of I-gel LMA and classic LMA on preoperative and postoperative acoustic voice analysis parameters, and VHI-30 scores were statistically evaluated.

Acoustic voice analysis

For voice recordings, an Audio-Technica AT 2005 model dynamic microphone (Audio-Technica Corporation, Tokyo, Japan) was used at a distance of 5 cm from the mouth in a room that is far from the hospital crowd where environmental noise is minimal. Recordings of all patients were performed as a mono sound recording at a sampling rate of 44,100 Hz and in a 16-bit sampling format. For acoustic voice analysis, the patients were asked to utter the vowel “a” for five seconds three times. An average of three utterances was recorded. In the acoustic voice analysis, F0, Jitter%, and Shimmer% were evaluated. All acoustic evaluations were performed online using Windows 7 operating system (Microsoft Corporation, Redmond, USA) via Praat (Paul Boersma, 2001, Version 6.017, http://www.praat.org/) program. Praat is an easy-to-use non-invasive computer program that can measure various aspects of sound. Spectrograms in Praat were used to evaluate the format frequencies.

Voice Handicap Index

The patients completed the “Voice Handicap Index” questionnaire [12] before and after the surgery for the subjective evaluation of voice. According to the responses on this scale, the degree of physical, social, and functional handicaps of the patients’ voice in daily life was determined. Each item is scored on a five-point scale (0-4). The total score is between 0 and 120. A high score indicates severe subjective voice impairment.

Statistical analysis

Statistics were performed using SPSS Statistics version 21.0 (IBM Corp, Armonk, USA). Descriptive statistics were presented as mean ± standard deviation (SD). Categorical variables were presented as percentages. The distribution of data was evaluated with the Shapiro-Wilks test. Chi-square test, Fisher's exact test, and Student’s t-test were used to compare demographic data of the groups. Paired t-test was used to analyze normally distributed continuous variables, and the Wilcoxon signed-rank test was used for normally distributed ones for pre-post operational data. Statistical significance was accepted for values of p < 0.05.

## Results

Between March 2019 and July 2019, a total of 88 patients were included in the study. When the groups were evaluated in terms of demographic data, the gender distribution of the patients was statistically similar between the groups (p > 0.05). The mean age of the I-gel group was 44.75 ± 12.68 years and the mean age of the classic group was 40.66 ± 13.20 years. No statistically significant difference was observed between the groups in terms of patient ages (p: 0.142). Smoking and alcohol use rates were similar among the study groups (p: 0.248, p: 0.494) (Table 1).

**Table 1:** Demographic data, smoking, and alcohol use of the groups ^a^ Chi-square test, ^b^ Fisher exact test, ^c^ Students t-test, SD: Standard deviation

	I-gel group (n = 44) Mean ± SD	Classic group (n = 44) Mean ± SD	p-value
Gender (Male/Female)	23 / 21	23 / 21	1.000^a^
Age (year)	44.75 ± 12.68	40.66 ± 13.20	0.142^c^
Smoking (%)	11 (% 25)	16 (% 36.4)	0.248^a^
Alcohol use (%)	2 (% 4.5)	0	0.494^b^

**Figure 1:**
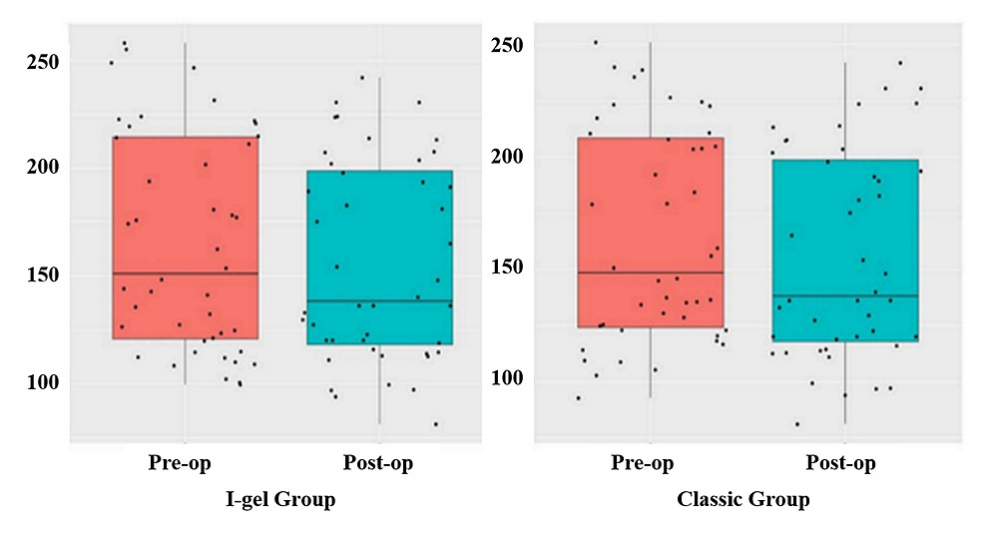
Box plot graphs of preoperative and postoperative F0 values of the groups Pre-op: Preoperative, Post-op: Postoperative

When the preoperative and postoperative F0, jitter, shimmer, and VHI-30 values in the I-gel and classic LMA groups were compared, the changes in the preoperative and postoperative F0, jitter, and VHI-30 values in the I-gel group were statistically significant (p: 0.002, p: 0.001, p < 0.001) (Table 2, Figure 1). Shimmer values were not significantly different (p: 0.762) (Table 2). In the classic LMA group, preoperative and postoperative F0, jitter, shimmer, and VHI-30 values were statistically significantly different (p: 0.001, p: 0.012, p: 0.036, p < 0.001) (Table 2, Figure 1).

**Table 2:** Preoperative and postoperative F0, jitter%, shimmer%, and VHI-30 values of the groups Wilcoxon signed-rank test. SD: Standard deviation. F0: Fundamental frequency VHI: Voice Handicap Index.

	I-gel group (n=44) Preoperative Mean ± SD	I-gel group (n=44) Postoperative Mean ± SD	p	Classical group (n=44) Preoperative Mean ± SD	Classical group (n=44) Postoperative Mean ± SD	p
F0	164.91 ± 50.02	155.90 ± 48.33	0.002	164.42 ± 46.95	156.03 ± 46.18	<0.001
Jitter (%)	0.50 ± 0.79	0.58 ± 0.69	0.001	0.37 ± 0.25	0.54 ± 0.41	0.012
Shimmer (%)	3.26 ± 1.57	3.20 ± 1.73	0.762	2.17 ± 1.51	2.67 ± 1.99	0.036
VHI-30	3.13 ± 3.59	6.27 ± 3.70	<0.001	3.27 ± 2.80	6.47 ± 4.88	<0.001

## Discussion

The results of the voice analysis performed in our study showed that I-gel LMA and conventional LMA negatively affect voice performance in the preoperative and postoperative periods. This situation was also observed in the VHI-30 index test, which is a subjective analysis. However, when this situation was evaluated in terms of shimmer analysis, the decrease in voice quality in the early postoperative period was more limited in the I-gel group. This study is unique considering the evaluation of classical LMA and I-gel LMA in terms of sound performance.

SAD is preferred especially in the outpatient setting because it is less traumatic compared to ETI and does not require muscle relaxants. This allows patients to recover quickly and to be discharged early [13,14]. Therefore, there are many SADs available, such as the enhanced classical LMA, ProSeal LMA, and I-gel LMA. A newer SAD, the I-gel airway, does not require an inflatable cuff and creates an adequate airway seal. As a result, it can minimize the risk of tissue compression and reduce the incidence of postoperative pharyngolaryngeal morbidity [4,5]. However, studies on the effect of these new SADs on sound quality are very limited. Although the larynx is the main structure in the formation of the voice, the supraglottic anatomical structures have an important effect on the quality of the voice. Traumatic situations that may develop in this region may cause negative effects on sound quality. Since SAD completely covers the pharynx, it may cause negative results in the anatomical structures of the pharynx unintentionally during anesthesia. Although this situation does not cause serious morbidity, the hoarseness that will occur in the postoperative period in the voice quality of the patients may cause both anxiety and deterioration in the quality of life [3,11]. It may be an advantage that I-gel LMAs are better suited to the pharyngeal anatomy and do not require an additional inflatable cuff to prevent leakage, as in conventional LMAs [15]. In our study, acoustics voice analysis performed in the first 48 hours postoperatively revealed significant deteriorations in the F0 and jitter% l values in both classic LMA and I-gel LMA groups, but there was no significant deterioration in the I-gel group in terms of shimmer % values. These results showed that the deterioration in voice quality may be more limited in the early postoperative period compared to classical LMA, although it is limited in the I-gel group. Prospective studies with larger series are needed on this subject.

Shimmer and jitter analyses are tests in terms of objectively showing changes in sound quality [16]. In the shimmer and jitter test, the level of increase compared to the preoperative period indicates deterioration in sound quality. This may be useful for SADs to predict involuntary trauma in the pharynx region. In our study, although the increase in the jitter test was significant in both groups compared to the preoperative values, its imperfection in the I-gel group and the decrease in the shimmer test values in the I-gel group indicate that the I-gel quite limited the trauma in the pharynx region. We think that this is important in terms of showing that I-gel limits the deterioration in sound quality.

The VHI-30 index test is one of the most reliable standardized self-evaluation assessments in the diagnosis of voice disorders. That tool is the most used in daily clinics [17]. The VHI-30 index test is also used in the evaluation of voice disorders after intubation [18]. However, we could not find any study on the evaluation of voice disorders after different SAD applications. In the present study, the VHI-30 index test showed similar results in terms of deterioration of voice quality in both groups. The similarity in the VHI-30 index test compared to other objective tests used in the study can be explained by the fact that this method is subjective and shows individual variability.

The present study had some limitations. This study single center. The effect of I-gel or classic LMA of different sizes in male and female patients on voice performance has not been investigated. The long-term consequence of voice performance was not evaluated.

## Conclusions

In conclusion, different SADs impair vocal performance in the first 48 hours postoperatively. However, this effect was limited in the I-gel group, suggesting that I-gel LMAs may be a good choice in patients for whom LMA use is appropriate. This may be even more important in patient groups where voice quality is needed. Comprehensive prospective studies to be conducted can guide in this regard.
